# Supplementary material for: Children’s views on research without prior consent in emergency situations: a UK qualitative study
Source: BMJ Open. 2018 Jun 9;8(6):e022894. doi: 10.1136/bmjopen-2018-022894 (PMC6009563; doi:10.1136/bmjopen-2018-022894)
Supplement: Supplementary file 1 [file bmjopen-2018-022894supp001.pdf]

Supplementary file 1: Description of RWPC in interviews

Normally, when doctors want to do research to find out the best medicine to give children to make them better they usually speak to mums, dads and children themselves to see if they would like to take part in the research. This is just to check that they are happy with the research before a medicine is given. But when children are very poorly and need emergency medicine- like in the back of an ambulance- there is no time to speak to anyone about the research. Doctors have special permission to give the medicine without asking parents and children first.

(Check child understands at this point and use drawing if necessary to assist understanding)

The reason this is allowed to give medicine as part of research without asking first is so that doctors can find out which are the best medicines to use to treat children when they are very sick. If they tried to speak to parents first it would mean that children wouldn't be given the medicine they need at the right time.
