# Supplementary material for: Children’s views on research without prior consent in emergency situations: a UK qualitative study
Source: BMJ Open. 2018 Jun 9;8(6):e022894. doi: 10.1136/bmjopen-2018-022894 (PMC6009563; doi:10.1136/bmjopen-2018-022894)
Supplement: Supplementary file 2 [file bmjopen-2018-022894supp002.pdf]

Supplementary file 2: vignette used in interviews

A little boy called Daniel had an illness which meant he sometimes had to go to hospital to be helped by the doctors and nurses to get better. The doctors asked Daniels mum and dad whether they could give him some different medicine to see if it was better than the medicine he usually got when he was unwell. They wanted to see what happened to Daniel when he got the new medicine and wanted to write extra information down about the new medicine. The new medicine was safe, but the doctors didn’t know whether it was better or the same as the medicine Daniel usually got. The doctors decided that because Daniel was feeling well again and had had some rest, they wanted to explain to Daniel about the different medicine and the reason why they wanted him to take part. Daniel is the same age as you.
