# Supplementary material for: Children’s views on research without prior consent in emergency situations: a UK qualitative study
Source: BMJ Open. 2018 Jun 9;8(6):e022894. doi: 10.1136/bmjopen-2018-022894 (PMC6009563; doi:10.1136/bmjopen-2018-022894)
Supplement: Supplementary file 3 [file bmjopen-2018-022894supp003.pdf]

Voices Project–Children's Views

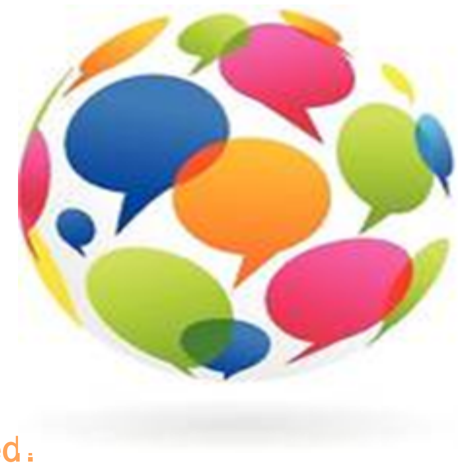

WOULD YOUR CHILD LIKE TO TAKE PART ?

What is the study about?

We are doing this study to find out what children think about research in emergency situations (such as in A&E).

When children are very sick it is important to give them medicine quickly. This means that there is no time to speak to parents or children about taking part in research before the emergency medicine is given. The doctors give the treatment first, and then speak to the parents about the study when their child is a bit better. We would like to know if children think it's ok to do research in emergencies without telling the parents or the child first.

Who can be involved?

We are interested to talking to children aged 7-<16 years old who have received emergency hospital treatment.

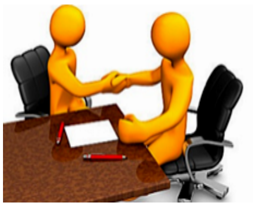

What is involved

We would like children to take part an interview with a researcher called Louise. Th can be at your home, on the phone, or at Alder Hey Children's Hospital; this is your choice. It will take about 45 minutes to an hour.

In the interview Louise will talk to your child about how research is done in emergencies. We would like to know what your child thinks about this type of research and find ways of involving children in decisions about emergency research in the future.

Following the study

We would like to give your child some vouchers (£10) as a thank you for participating in our research study and a certificate of participation.

Who is involved in the study?

The study is being organised by the Universit of Liverpool and has been approved by a research ethics committee (ref: 15/NW/0915).

If you would like to be involved please speak to your child and contact Louise sending her your contact details and age of the child/ young person so we can send out the correct participant information sheet (the information in these varies with the age of the child)
t: 0151 795 5326
E: consent@liv.ac.uk

And, check out our website:
http://www.liv.ac.uk/psychology-health-and-society/research/childrens-voices/about/
