# Supplementary material for: Children’s views on research without prior consent in emergency situations: a UK qualitative study
Source: BMJ Open. 2018 Jun 9;8(6):e022894. doi: 10.1136/bmjopen-2018-022894 (PMC6009563; doi:10.1136/bmjopen-2018-022894)
Supplement: Author's manuscript [file bmjopen-2018-022894.draft_revisions.pdf]

BMJ Open

BMJ Open is committed to open peer review. As part of this commitment we make the peer review history of every article we publish publicly available.

When an article is published we post the peer reviewers' comments and the authors' responses online. We also post the versions of the paper that were used during peer review. These are the versions that the peer review comments apply to.

The versions of the paper that follow are the versions that were submitted during the peer review process. They are not the versions of record or the final published versions. They should not be cited or distributed as the published version of this manuscript.

BMJ Open is an open access journal and the full, final, typeset and author-corrected version of record of the manuscript is available on our site with no access controls, subscription charges or pay-per-view fees (http://bmjopen.bmj.com).

If you have any questions on BMJ Open's open peer review process please email info.bmjopen@bmj.com

BMJ Open

CHILDREN SUPPORT RESEARCH WITHOUT PRIOR CONSENT IN EMERGENCY SITUATIONS: A QUALITATIVE STUDY

Journal:	*BMJ Open*
Manuscript ID	bmjopen-2018-022894
Article Type:	Research
Date Submitted by the Author:	22-Mar-2018
Complete List of Authors:	Roper, Louise; University of Liverpool, Institute of Psychology Health and Society<br>Sherratt, Frances C.; University of Liverpool, Department of Psychological Sciences<br>Young, Bridget; University of Liverpool, Department of Psychological Sciences<br>McNamara, Paul; University of Liverpool, Department of Child Health, Institute of Translational Medicine<br>Dawson, Angus; The University of Sydney Camperdown Campus<br>Appleton, Richard; Alder Hey Children's NHS Foundation Trust, Neurology<br>Crawley, Esther; University of Bristol, Bristol Medical School<br>Frith, Lucy; University of Liverpool, Department of Health Service Research<br>Gamble, Carrol; The University of Liverpool, Biostatistics<br>Woolfall, Kerry; University of Liverpool, Institute of Psychology Health and Society
Keywords:	ACCIDENT & EMERGENCY MEDICINE, Paediatric intensive & critical care < INTENSIVE & CRITICAL CARE, MEDICAL ETHICS, QUALITATIVE RESEARCH, Consent

SCHOLARONE™
Manuscripts

TITLE: CHILDREN SUPPORT RESEARCH WITHOUT PRIOR CONSENT IN EMERGENCY SITUATIONS: A QUALITATIVE STUDY

Louise Roper[1], Frances Sherratt[1], Bridget Young[1], Paul McNamara[2], Angus Dawson[3], Richard Appleton[4], Esther Crawley[5], Lucy Frith[1], Carrol Gamble[6], Kerry Woolfall[1].

Corresponding author: Dr Kerry Woolfall, Institute of Psychology, Health & Society, University of Liverpool, Liverpool, UK, K.Woolfall@liverpool.ac.uk, Tel: 0151 794 4634

Affiliations:

1. Institute of Psychology, Health & Society, University of Liverpool, Liverpool, UK

2. Department of Child Health, Institute of Translational Medicine, University of Liverpool, Liverpool, UK

3. School of Public Health, University of Sydney, Sydney, Australia

4. Neurology Department, Alder Hey Children's Hospital, Liverpool, UK

5. Bristol Medical School, University of Bristol, Bristol, UK

6. Clinical Trials Research Centre (CTRC), University of Liverpool, Liverpool, UK

Key words: Accident & Emergency Medicine, intensive & critical care, medical ethics, qualitative research, consent

Word count: 3935

For peer review only

ABSTRACT

Objectives: We explored children's views on research without prior consent and sought to identify ways of involving children in research discussions.

Design: Qualitative interview study.

Setting: Participants were recruited through a UK children's hospital and online advertising.

Participants: 16 children aged 7-15 years with a diagnosis of asthma (n=14) or anaphylaxis (n=2) with recent (< 12 months) experience of emergency care.

Results: Children were keen to be included in medical research and viewed research without prior consent as acceptable in emergency situations if trial interventions were judged safe. Children trusted that doctors would know about their trial participation and act in their best interests. All felt that children should be informed about the research following their recovery and involved in discussions with a clinician or their parent(s) about the use of data already collected, as well as continued participation in the trial (if applicable). Participants suggested methods to inform children about their trial participation including an animation.

Conclusions: Children supported, and were keen to be involved in, clinical trials in emergency situations. We present guidance and an animation that practitioners and parents might use to involve children in trial discussions following their recovery.

Strengths and limitations of this study

- This is the first study to explore the views and acceptability of research without prior consent (RWPC) among children with experience of receiving emergency treatment for life threatening conditions.
- We provide guidance and an animation to assist trial practitioners in involving children in RWPC discussions in collaboration with parents.
- Participants did not have personal experience of RWPC so findings were limited to children's views on hypothetical scenarios.
- Participants lived in areas with varied levels of social deprivation. Despite attempts to further maximize sample diversity by use social media, the majority of children were recruited from one UK children's hospital.

INTRODUCTION

Informed consent for participation in research is a key principle of good clinical practice and protects an individual's right to autonomy[1 2]. Children under the age of 16 are not legally permitted

For peer review only

to give consent for their participation in clinical trials of drug treatments[3]. Nevertheless, the United Nations Convention on the Rights of the Child[4], international legislation[5] and research guidelines[6] recommend that children should be involved in decision making processes in a way that is age appropriate to the child and the context of their family. It is recommended that assent is sought from children before they take part in research, where informed consent is not possible, although the role of assent has been criticized for being unclear in research discussions with children.[7 8]

Neither informed consent nor assent are appropriate or feasible in acute paediatric emergency situations[9], as children are incapacitated and the time taken to discuss research with parents could delay lifesaving treatments[10 11]. To facilitate crucial clinical research and to advance evidence-based emergency medicine[12], changes to international legislation have allowed doctors to enter patients into clinical research without prior consent (RWPC)[3 13-16]. In the United Kingdom, RWPC, also known as deferred consent, involves approaching parents or legal representatives after the investigational treatment has been given and seeking permission for the use of data already collected, as well as any continued participation in the trial[17].

Guidance[18] and legislation[15] state that where possible, children should be involved in RWPC discussions once they have recovered. However, practitioners report that RWPC discussions with parents take place at the bedside, shortly after the emergency situation has passed and that children are rarely involved at this point because they too sick[19]. It is therefore likely that most children randomised into UK trials conducted in time critical situations have no knowledge or involvement in the decision about their participation in emergency research.

Despite growing literature on the views of parents and practitioners regarding paediatric trials conducted in life threatening situations[18 20 21], the opinions of children have not been investigated. This is an important omission as it is they who are most directly affected in this process. We aimed to explore children's perspectives on paediatric emergency research, including their views on the acceptability of RWPC and ways of involving them in RWPC discussions when they have recovered.

METHODS

Study design and setting

We chose a qualitative interview design to help us explore children's views in a flexible, child friendly way, and to provide insight into children's perspectives on RWPC[22 23].

We recruited children for interviews through a children's hospital in the North West of England, supplemented by online-advertising to promote sample diversity.

For peer review only

Patient and Public Involvement

Six members of the National Institute for Health Research (NIHR) Young Person's Advisory Group (YPAG) joined our 'Voices Advisory Group' to inform all aspects of the study. This included their collaboration in the development of recruitment materials, a topic guide, which included age-appropriate explanations of key topics (e.g. RWPC; see Supplementary file 1) and vignette (see Supplementary file 2). KW and LR discussed initial findings with the Voices Advisory Group to assist analysis and interpretation. Members of the Voices Advisory Group also presented the findings with KW to a wide audience (at conferences, and University events)

Selection of participants

Children were eligible if they: were aged 7-15 years; had written parental/legal representative consent to participate; had received emergency treatment in hospital in the previous 12 months; had capacity to assent and an acceptable standard of English.

Two clinical practitioners identified and approached parents and children who met the inclusion criteria, explained the aims of the study and provided age appropriate information sheets. Parents of the children who wished to participate completed a contact form, which was posted to LR. For online recruitment, FS contacted relevant support groups and asked them to place a study advertisement (Supplementary file 3) on their Facebook page, Twitter account, or website. The advert included details of how children or parents could contact the study team to register interest in participation.

Interview design and conduct

LR arranged interviews either in the family's home, hospital or by telephone depending on participant preference. Before interviews LR explained the study, referring to the information sheets and consent/assent forms provided. Consent and assent forms were completed by parents and children before the interview began.

We began interviews by showing children the Nuffield Council's animation[6] on a laptop or iPad to help set the scene and explain clinical research participation in a child-friendly way. No child had previous experience of RWPC, although they all had experienced at least one episode of receiving emergency care. We presented RWPC neutrally so that children could give their views freely. We also reassured children that there were no right or wrong answers.

LR conducted all interviews. Recruitment and interviews were discontinued when data saturation was reached, i.e. the point where no new themes were discovered in the analysis[24].

For peer review only

Interviews were audio-recorded, transcribed verbatim, and then anonymised. Participants were given a £10 shopping voucher and a certificate of participation as appreciation for their time.

Data analysis

Our approach to data analysis was interpretive and iterative, referring back and forth between developing analysis and gathering new data for evidence of children's views on research in emergency situations, the acceptability of RWPC and ways of involving children in discussions about research in this context[25 26]. A key consideration during analysis was that of catalytic validity, whereby findings should be relevant to future research and practice[29]. NVivo software[30] assisted data organisation. Several members of the research team (KW, LR, FS, BY) contributed to the analysis to ensure analytical rigor[26 28]We present selected interview quotations (with pseudonyms) that illustrate research themes across a range of participants. Where quotes have been shortened for brevity or to remove identifiable information, omitted text is marked with '…', and explanatory text is in brackets.

RESULTS

Participants

Sixteen children (aged 7-15 [mean 10.2] years) were interviewed. At the children's request, parents were present for most the interviews (n = 12/16, 75%). Most participants had chronic asthma (n = 14/16, 87%) and were recruited by a paediatric doctor (PM) in an outpatient clinic or hospital ward (n=2/16, 13%). Two participants suffered with anaphylaxis and were recruited via Facebook. Interviews averaged 34 minutes in length, ranging from 21-57 minutes (Table 1). Participants' postcodes indicated that 9/16 (56%) participants lived in areas of high deprivation (Indices of Multi Deprivation [IMD] decile 1-3), 5/16 (31%) lived in areas of moderate deprivation (IMD decile 4-6), whilst 2/16 (13%) children lived in the least deprived (IMD decile 7-10) areas of the UK[31].

Pseudonym	Gender	Age	Health condition	Parent present
Chloe	Female	12	Asthma	Yes
Charlie	Male	7	Asthma	Yes
James	Male	11	Asthma	Yes
Emily	Female	15	Asthma	No

For peer review only

Niamh	Female	15	Asthma	Yes
Kaitlin	Female	8	Asthma	Yes
Joseph	Male	7	Asthma	Yes
Josh	Male	11	Asthma	Yes
Mia	Female	9	Asthma	Yes
Daisy	Female	7	Asthma	Yes
Kevin	Male	8	Asthma	Yes
Patrick	Male	9	Asthma	No
Lola	Female	7	Asthma	Yes
Ryan	Male	8	Asthma	Yes
Tom	Male	15	Anaphylaxis	No
Tilly	Male	15	Anaphylaxis	No

Table 1: Participant and interview details

Support for RWPC, but only if a trial treatment was judged safe

All children voiced support for RWPC in emergency situations where a child's life was in danger, such as during an asthma attack or seizure. They explained that while parents and children should usually be asked for "permission" before the research takes place, RWPC was acceptable in emergency research as it is important that doctors were able to give trial medicines without any delay in this situation.

> **Chloe, aged 12:** *most of the time (in research) you should ask the permission from the parents and the children… I think they can do that (research) without asking for permission because if it's an emergency and you have to give the medicine.*

The researcher (and the vignette of Daniel, see S3), informed children that only medicines that were believed to be safe would be used in paediatric trials. Children viewed this information as important, indeed, many stated that RWPC was only acceptable if doctors thought the treatment being tested was safe.

> **Josh, aged 11:** *If, say, like you're giving them a drug that hasn't been proven to have high probability of being safe, I don't think it would be good to give it them, but if it was probably going to be safe for them, I think they'd be fine.*

For peer review only

One child supported RWPC in the hope that the treatment being tested would help children to recover more quickly, and to inform future paediatric medicine. However, he warned that parents may have a different perspective and may be angry to find out that their child had been entered into a trial without informed consent if it turned out that the treatment was not effective.

Researcher: *So what do you think about doing research without asking parents or children when they're very sick?*

James aged 11: *I think the parents would be a bit ticked off like because they just want to get their child better, like I know my mum definitely would be…I would like it because if that drug did work on the off-chance then I would want to tell people* (other children with chronic health conditions).

Misconceptions and misunderstandings

Many children appeared to understand that trials *"try to see if"* the medicine being investigated makes children *"better or not"* (Lola aged 7). However, a few children appeared to hold a misconception that research participation would have similar benefits to clinical care. For some children this misconception seemed to be linked to the involvement of clinicians in the trial. Children stated they would not be upset about being entered into a RWPC trial as long as *"the doctors know"* (Emily aged 15) about their participation. As the quotes below suggest, children trusted clinicians to have their best interests at heart and-wanted assurance that doctors and nurses would know about, and have approved, their involvement in a trial.

Researcher: *So what if they gave you a new type of nebuliser and not told you? Do you think if it's that type of thing you should have been asked* (about beforehand)*, or do you still think it's* (RWPC is) *okay?*

Emily aged 15: *The doctors know* (about being given a trial intervention)*?*

Researcher: *The doctors yes.*

Emily aged 15: *Oh yes, definitely.*

Researcher: *Would you be upset that they have tried something on you and not asked you first?*

Ryan aged 8 *No.*

Researcher: *Why is that?*

Ryan aged 8: *Because the doctors and the nurse, they know.*

Others believed that within a trial, children would receive the 'right' treatment, whist another implied that RWPC would only be acceptable if the intervention was effective:

Daisy aged 7: *"He'll (Daniel) get the right medicine to make him feel better.*

Tilly aged 14: *I don't see the problem if it's going to stop it* (anaphylaxis).

Why children want to be informed and involved in RWPC discussions

Children felt strongly they should be involved in RWPC discussions when they were feeling better. Several asked rhetorically, "Why shouldn't we be told?" when asked about their participation in research. Irrespective of their age, children reasoned they should be informed about the treatment received in a trial because *"it's going to impact on their body"* (Tilly, aged 14) not their parents' bodies. Indeed. Tilly added that not being informed about their participation in a trial would *"feel like I'd been sort of left out, like my opinions and views sort of, I don't get to express them"* suggesting that not being included in RWPC discussion would deny children their voice and the opportunity to have a say in their healthcare treatment.

A few children described wanting to be informed about their involvement in research and the trial results to *"know what the trial had proved"* (Chloe aged 12) for their own chronic health condition. They expressed a wish to help inform future knowledge about treatments for other children by finding "*out about new medicines on the computer that are better than other ones and then they could give us it and then it might make us better."* (Joseph aged 7).

Finally, two young boys (<12 years) wanted to know about their participation because they viewed medical research as exciting. For them children should be informed about their participation in research because "*it could be fun to take part in stuff"* (Joseph, aged 7) and *"it's quite cool to be part of like a scientific medical thing"* (Josh, aged 11).

For peer review only

Who should explain RWPC to children?

Most children commented that the best person to explain that they had participated in a study while they were ill would be a doctor or nurse as they would be the most knowledgeable.

> **Tilly, aged 14:** *if it's a nurse who knows all about it and then also has studied kids, they can then help with that as well, help them do it, understand what's happened and go through it. But I don't think I'd listen if my mum told me. I would want it more, the medical advice really.*

> **Kaitlin, aged 8**: *By the doctor … because he can explain it more.*

Some envisaged they would have questions about the research which they would like a doctor or nurse to answer, such as: "*How could it affect me? What would be like the aftermath of it?"* (James aged 11), "*What could the risks be?" (*Joseph aged 7) as the doctor or nurse would be best placed to answer.

Although some children said they *"wouldn't mind who told"* (Chloe aged 12) them about their participation in a trial, a few younger children stated that they would prefer to be informed by their mothers because it may *"feel a bit awkward"* talking to the doctors and its *"easier with my mum"* (Mia aged 9).

Children's role in decisions about the use of their information

Children varied in their views about the role of children in RWPC decisions when they were feeling better. Younger children were typically happy for their parents (mainly mothers) to make the final decision about the use of their information in a trial.

> **Kevin aged 8**: *Up to my mum.*

> **Joseph, aged 7**: *I would listen to my mum and the doctors because older people can sometimes be cleverer than younger people than them, because they've had more years to learn about stuff.*

Participants of all ages echoed this by commenting that children younger than themselves, *"don't really understand […] what the research is about"* (Chloe aged 12) and *"might be a bit too*

For peer review only

young" (Joseph aged 7) to make such decisions, and that for such children it should be a parent's role to make a decision about the use of their information for research purposes.

Similarly, some children stated that they would accept their parents' decision, even if it was at odds with their own views, whilst others wanted to share in decision-making with their mothers.

> **Emily aged 15:** *My little brother, he's 11 and I think he's sensible so he'd probably choose the right thing. But if you're a bit younger I think it should be up to your mum.*
>
> **James, aged 11:** *If mum said no and I said yes, I would just listen to mum.*
>
> **Researcher:** *What about if you wanted to be in it but mum said no, then who should (the doctor) listen to?*
>
> **Chloe, aged 8:** *Both of us.*

In contrast, a few teenage participants felt that *"they should be able to actually make a medical choice for themselves because it's impacting them in the end"* (Tilly aged 14). Therefore, their decision about the use of their data and involvement in any follow up, should override, or at least have greater value, than their parents' decisions.

> **Researcher:** *What if your mum says yes, that's fine and you say no? Then who should be in charge?*
>
> **Emily aged 15:** *Me*

Child friendly resources to help communicate RWPC: introducing the 'You took part in Research animation'

Finally, we sought participants' suggestions on the most appropriate resources to help to engage with children in RWPC discussions when they were feeling better. Participants emphasized the need for a face to face discussion so doctors or nurses could *"explain it to you, or give you a leaflet, then…you can ask questions in person"* (Chloe aged 12). While a few suggested that leaflets, websites and picture books would be useful in explaining RWPC, most favoured an online animation that could be used either in hospital as part of a face to face discussion, or *"at some point when I was at home"* (Tom aged 13) to *"make sure they understand everything properly"* (Josh aged 11) to explain RWPC.

For peer review only

Patrick aged 9: "*because some doctors don't know how far to explain stuff when they are particularly young kids. So even the doctors to have on their phone or their iPad and go this is what we've done, and then they watch it and then they explain a bit more then*".

The findings of this study encouraged the study team to develop an animation with the help of the Voices Advisory Group and three of interviewees in the current study who wished to help with this. The 'You took part in research' animation available to view on YouTube (https://www.youtube.com/watch?v=_Fs1yUxeBFQ), focuses on explaining why consent could not be sought before a child took part in research.

DISCUSSION

We believe this is the first study to explore the views and acceptability of RWPC among children with experience of receiving emergency treatment in hospital. The children we interviewed supported RWPC in the hope that such research would contribute to developments in children's emergency medicine for the benefit of themselves and other children. However, RWPC was only acceptable to children where trial interventions were believed by the clinicians caring for them to be safe. This finding also echoes previous literature on therapeutic misconceptions about trial participation amongst adults[34], as some children held the misconception that research participation would have similar benefits to clinical care. Others appeared to trust that doctors would know about their trial participation and act in their best interests. Others believed that within a trial, children would receive the 'right', treatment. Such findings suggest that trial information materials and practitioner RWPC discussions should clearly explain that doctors don't know which intervention is the best, which is why a trial is needed. This information should be supplemented with details of other treatments given (if applicable) and any additional monitoring that occurred.

This study has strengths and limitations. This is the first study to explore the views and acceptability of RWPC among children with experience of receiving emergency treatment in hospital. We strengthened our qualitative sampling by conducting interviews until no new relevant knowledge was obtained from new participants (data saturation). All participants had a chronic health condition and experienced multiple hospital admissions for emergency lifesaving treatments. However, our findings do not reflect the views of acutely ill children admitted to hospital for the first time. Participants did not have personal experience of emergency medicine research so findings were limited to children's views on hypothetical scenarios about RWPC. Finally, the study findings are mainly limited to the views of children recruited from an asthma clinic in one UK children's hospital. Nevertheless, there was variation in the sample, as children lived in areas with differing

levels of social deprivation. Despite the successful use of websites and social media advertising to recruit parents to similar qualitative studies[21 32], only two children responded to social media advertisement. The low response to this indicates social media such as Facebook and Twitter may not be an effective route for recruiting children to research. Placing adverts on social media more commonly used by children (e.g. Snapchat)[33] may have been more successful.

Children's support of research without prior consent in emergency situation reflects the findings of previous studies on this topic involving trial practitioners and parents[18 19 21 32 35]. Where studies[18 32 36] have shown that parents can have initial negative responses to RWPC, children did not appear to be shocked or surprised at this alternative to giving informed consent before the research intervention. This may reflect their lack of familiarity with standard research consent processes, or indeed, their lack of previous involvement in decisions about their healthcare. Our findings suggest that children trust doctors and parents to make appropriate decisions about their participation in research when they are unable to do so themselves. However, children were clear that when they were feeling better, adults (particularly mothers and doctors) should inform them of their trial participation and discuss the use of their data.

Children in our study acknowledged the important role that children, parents and doctors played in research decision making. This reflects guidelines recommending that decisions about research should involve discussion and information exchange between children, their parents and doctors[6 12 37-40]. Some described the prospect of research participation as exciting and fun, whilst others, seemed to want the satisfaction of knowing if they had contributed to research that might improve future treatments for both themselves and others. Children wanted to be involved in decisions regarding their own healthcare [41 42]. Younger children were happy for their parents to take the lead or a shared role in research decision-making. A few teenagers felt their views should carry greater weight than their parents suggesting that practitioners should be aware of the possibility that teenagers may wish to take a lead in such decisions. As previous literature [19 21 32] and Voices study findings all show support for RWPC, we do not anticipate divergence in child and parent views on consent in this situation; mainly because the intervention has already been given and children have commonly recovered when research discussions take place.. The challenge is ensuring children are given the opportunity to be (retrospectively) informed about their research participation and actively involved in research discussions and decision making with their parents.

We acknowledge there may be practical barriers to involving children in RWPC discussions, as they may not be well enough, or have the capacity to be involved before discharge from hospital. Nevertheless, our findings[19] indicate that when they have recovered children are not given the opportunity to know they have participated in research or a say in whether their data continue to be

For peer review only - http://bmjopen.bmj.com/site/about/guidelines.xhtml

used in research. Based on the accounts of the children we interviewed, this is unlikely to be acceptable to children who participate in trials to evaluate interventions used in emergency care. As one child we interviewed eloquently stated, *"it's not impacting their life, it's impacting your own"* (Tilly, aged 15).

Box 1 (guidance to inform and involve children in RWPC discussions) provides an update to CONNECT study RWPC guidance[43], which aims to assist trial practitioners in involving children in RWPC discussions in collaboration with parents. To respect the wishes of children we encourage trial staff to involve them in trial discussions in hospital if they are well enough. The 'You took part in research' animation can be used to support these discussions. If this is not possible before children are discharged from hospital. Age appropriate information can be provided to facilitate family discussions about trial participation at home, including contact details if children wish to discuss their participation with the trial team Whilst our focus in this paper has been children (under 16 years) we believe our findings also relate to trial discussions with young people (16-18 years).

Children should be given an opportunity to be involved in making decisions about the use of their data in the trial and their continued enrolment if they have capacity.

- When children cannot be involved in trial consent discussions due to a lack of capacity, provide age appropriate trial information (such as a leaflet or website) to parents for children to read when they have recovered.
- Include a link to the 'You took part in research animation' https://www.youtube.com/watch?v=_Fs1yUxeBFQ) to help explain why consent could not be sought before a child took part in research.
- Provide research team contact details so that parents or children can discuss any aspect of the trial at a later date.

Box 1: Guidance to inform and involve children in RWPC discussions

Further research is needed to explore the experiences of children with first-hand experience of RWPC and associated discussions.

Transparency statement

The lead author affirms that the manuscript is an honest, accurate, and transparent account of the study being reported; that no important aspects of the study have been omitted; and that any discrepancy from the study as originally planned has been explained. The report is independent

research supported by the National Institute for Health research (Senior Research Fellowship, Prof Esther Crawley, SRF-2013-06-013). The views expressed in this publication are those of the authors and not necessarily those of the NHS, the National institute for Health Research or the Department of Health.

Author contributions: KW conceived the study. KW, BY, LR, and FS designed the study. PM, LR and FS recruited children to the study. LR conducted the interviews. LR, FS and KW analysed the data. KW and LR drafted the paper. All authors contributed substantially to its revision. KW takes responsibility for the paper as a whole. We would like to thank Beth Morris and Paige Karadag for their comments on the final draft.

Funding statement
The study was funded by the MRC Hubs for Trials Methodology Research (project R42) and supported by Wellcome Trust ISSF award.

Ethics statement
A National Health Service Research Ethics Committee (15/NW/0915) provided ethical approval.

Data sharing statement No additional data are available

References

1. Nelson RM, Beauchamp T, Miller VA, et al. The Concept of Voluntary Consent. *The American Journal of Bioethics* 2011;11(8):6-16. doi: 10.1080/15265161.2011.583318
2. Beauchamp T, Childress J. Principles of Biomedical Ethics. Oxford: Oxford University Press 2001.
3. Legislation.gov.uk. Medicines for Human Use (Clinical Trials) Regulations 10: SI 2004/1031 2004.
4. United Nations. Convention on the Rights of the Child. 1989. http://www.ohchr.org/Documents/ProfessionalInterest/crc.pdf (accessed 3rd June 2016).
5. legislation.gov.uk. Children Act 1989, 1989.
6. Nuffield Council on Bioethics. Children and clinical research: ethical issues. London: Nuffield Council on Bioethics, 2015.
7. Baines P. Assent for children's participation in research is incoherent and wrong. *Archives of Disease in Childhood* 2011;96(10):960-62. doi: 10.1136/adc.2011.211342
8. Madden L, Shilling V, Woolfall K, et al. Questioning assent: how are children's views included as families make decisions about clinical trials? *Child: Care, Health and Development* 2016;42(6):900-08. doi: 10.1111/cch.12347
9. Eltorki M, Uleryk E, Freedman SB. Waiver of informed consent in pediatric resuscitation research: a systematic review. *Acad Emerg Med* 2013;20(8):822-34. doi: 10.1111/acem.12180 [published Online First: 2013/09/17]
10. DIRECTIVE 2001/20/EC OF THE EUROPEAN PARLIAMENT AND OF THE COUNCIL of 4 April 2001on the approximation of the laws, regulations and administrative provisions of the Member States relating to the implementation of good clinical practice in the conduct of clinical trials on medicinal products for human use. 2001; L 121/34. http://www.eortc.be/services/doc/clinical-eu-directive-04-april-01.pdf.
11. Roberts I, Prieto-Merino D, Shakur H, et al. Effect of consent rituals on mortality in emergency care research. *The Lancet* 2011;377(9771):1071-72. doi: http://dx.doi.org/10.1016/S0140-6736(11)60317-6
12. Brierley J, Larcher V. Emergency research in children: Options for ethical recruitment. *Journal of Medical Ethics* 2011;37(7):429-32.

For peer review only

13. Kompanje EO, Maas AR, Menon D, et al. Medical research in emergency research in the European Union member states: tensions between theory and practice. *Intensive Care Medicine* 2014;40(4):496-503. doi: 10.1007/s00134-014-3243-6
14. Iwanowski P, Budaj A, Czlonkowska A, et al. Informed consent for clinical trials in acute cirinary syndromes and stroke following the European clinical trials directive: investigators' experiences and attitudes. *Trials* 2008;9(45):1-6.
15. Legislation.gov.uk. The Medicines for Human Use (Clinical Trials) and Blood Safety and Quality (Amendment) Regulations 2008 941. 10. 2008
16. Council of the European Union. Proposal for a Regulation of the European Parliament and of the Council on Clinical trials on medicinal products for human use, and repealing Directive 2001/20/EC. 2013. (accessed 29/01/14).
17. Fost N, Robertson J. Deferring Consent with Incompetent Patients in an Intensive Care Unit. *IRB: Ethics and Human Research* 1980;2(7):5-6. doi: 10.2307/3564363
18. Woolfall K, Frith L, Dawson A, et al. 15 minute consultation: an evidence-based approach to research without prior consent (deferred consent) in neonatal and paediatric critical care trials. *Archives of disease in childhood - Education & practice edition* 2015;101(1):49-53. doi: 10.1136/archdischild-2015-309245
19. Woolfall K, Frith L, Gamble C, et al. How parents and practitioners experience research without prior consent (deferred consent) for emergency research involving children with life threatening conditions: a mixed method study. *BMJ Open 2015*;5(9):e008522 doi: 10.1136/bmjopen-2015-008522
20. Lyttle MD, Gamble C, Messahel S, et al. Emergency treatment with levetiracetam or phenytoin in status epilepticus in children—the EcLiPSE study: study protocol for a randomised controlled trial. *Trials* 2017;18(1):283. doi: 10.1186/s13063-017-2010-8
21. Woolfall K, Young B, Frith L, et al. Doing challenging research studies in a patient-centred way: a qualitative study to inform a randomised controlled trial in the paediatric emergency care setting. *BMJ Open* 2014;4(5) doi: 10.1136/bmjopen-2014-005045
22. Murphy E, Dingwall R. Qualitative methods in health services research. *Health services research methods* 1998:129 - 38.
23. Morrow V. Using qualitative methods to elicit young people's perspectives on their environments: some ideas for community health initiatives. *Health Education Research* 2001;16(3):255-68. doi: 10.1093/her/16.3.255
24. Guest G, Bunce A, Johnson L. How Many Interviews Are Enough?: An Experiment with Data Saturation and Variability. *Field Methods* 2006;18(1):59-82. doi: 10.1177/1525822x05279903
25. Strauss A, Corbin J. Basics of qualitative research: Techniques and procedures for developing grounded thoery 2nd edition. Thousand Oaks, CA: Sage 1998.
26. Mays N, Pope C. Assessing quality in qualitative research. *BMJ* 2000;320(7226):50-52. doi: 10.1136/bmj.320.7226.50
27. Braun V, Clarke V. Using thematic analysis in psychology. *Qualitative Research in Psychology* 2006;3(2):77-101. doi: 10.1191/1478088706qp063oa
28. Tong A, Sainsbury P, Craig J. Consolidated criteria for reporting qualitative research (COREQ): a 32-item checklist for interviews and focus groups. *International Journal for Quality in Health Care* 2007;19(6):349-57. doi: 10.1093/intqhc/mzm042
29. Stiles WB. Evaluating qualitative research. *Evidence Based Mental Health* 1999;2(4):99-101. doi: 10.1136/ebmh.2.4.99
30. NVivo qualitative data analysis Software [program], 2012.
31. Department for Communities and Local Government. File 7: all ranks, decile and scores for the indices of deprivation, and population denominators. In: Official Statistics, ed. London, 2015.

For peer review only

32. O'Hara CB, Canter RR, Mouncey PR, et al. A qualitative feasibility study to inform a randomised controlled trial of fluid bolus therapy in septic shock. *Archives of Disease in Childhood* 2017 doi: 10.1136/archdischild-2016-312515
33. Ofcom. Children and Parents: Media Use and Attitudes Report. London, 2017.
34. Lidz CW, Appelbaum PS, Grisso T, et al. Therapeutic misconception and the appreciation of risks in clinical trials. *Social Science & Medicine* 2004;58(9):1689-97. doi: https://doi.org/10.1016/S0277-9536(03)00338-1
35. Gamble C, Hickey H, Snape D, et al. Parental attitudes to deferred consent in randomised trials in emergency resuscitation: a postal survey of Meningitis Research Foundation members. *Submission pending* 2010
36. Woolfall K, Frith L, Gamble C, et al. How parents and practitioners experience research without prior consent (deferred consent) for emergency research involving children with life threatening conditions: a mixed method study. *BMJ Open* 2015;5(9) doi: 10.1136/bmjopen-2015-008522
37. Modi N, Vohra J, Preston J, et al. Guidance on clinical research involving infants, children and young people: an update for researchers and research ethics committees. *Archives of Disease in Childhood* 2014 doi: 10.1136/archdischild-2014-306444
38. Nuffield Council on Bioethics. Children and clinical research: ethical issues. London, 2015.
39. Paediatrics RCo, Committee CHEA. Guidelines for the ethical conduct of medical research involving children. *Archives of Disease in Childhood* 2000;82(2):177-82. doi: 10.1136/adc.82.2.177
40. Helsinki WEU-Do. World Medical Association Declaration of Helsinki. In: Assembly WG, ed. Fortaleza, Brazil, 2013.
41. Moore L, Kirk S. A literature review of children's and young people's participation in decisions relating to health care. *J Clin Nurs* 2010;19(15-16):2215-25. doi: 10.1111/j.1365-2702.2009.03161.x [published Online First: 2010/07/28]
42. Shilling V, Williamson PR, Hickey H, et al. Processes in recruitment to randomised controlled trials of medicines for children (RECRUIT): a qualitative study. *Health Technol Assess* 2011;15(15):1-116. doi: 10.3310/hta15150 [published Online First: 2011/03/30]
43. Woolfall K, Frith L, Dawson A, et al. Research without prior consent (deferred consent) in trials investigating the emergency treatmemt of critically ill children: CONNECT study guidance. Liverpool: University of Liverpol, 2015.

For peer review only

Supplementary file 1: Description of RWPC in interviews

Normally, when doctors want to do research to find out the best medicine to give children to make them better they usually speak to mums, dads and children themselves to see if they would like to take part in the research. This is just to check that they are happy with the research before a medicine is given. But when children are very poorly and need emergency medicine- like in the back of an ambulance- there is no time to speak to anyone about the research. Doctors have special permission to give the medicine without asking parents and children first.

(Check child understands at this point and use drawing if necessary to assist understanding)

The reason this is allowed to give medicine as part of research without asking first is so that doctors can find out which are the best medicines to use to treat children when they are very sick. If they tried to speak to parents first it would mean that children wouldn't be given the medicine they need at the right time.

For peer review only

Supplementary file 2: vignette used in interviews

A little boy called Daniel had an illness which meant he sometimes had to go to hospital to be helped by the doctors and nurses to get better. The doctors asked Daniels mum and dad whether they could give him some different medicine to see if it was better than the medicine he usually got when he was unwell. They wanted to see what happened to Daniel when he got the new medicine and wanted to write extra information down about the new medicine. The new medicine was safe, but the doctors didn't know whether it was better or the same as the medicine Daniel usually got. The doctors decided that because Daniel was feeling well again and had had some rest, they wanted to explain to Daniel about the different medicine and the reason why they wanted him to take part. Daniel is the same age as you.

For peer review only

Voices Project– Children's Views

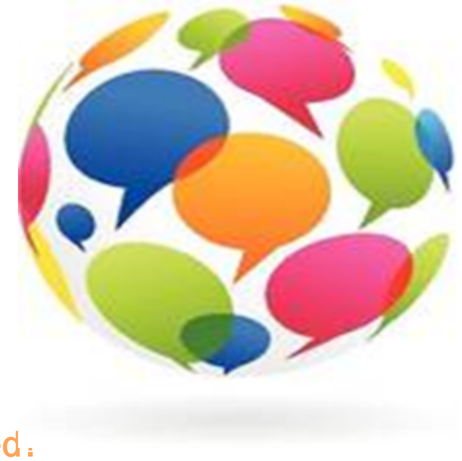

WOULD YOUR CHILD LIKE TO TAKE PART ?

What is the study about?

We are doing this study to find out what children think about research in emergency situations (such as in A&E).

When children are very sick it is important to give them medicine quickly. This means that there is no time to speak to parents or children about taking part in research before the emergency medicine is given. The doctors give the treatment first, and then speak to the parents about the study when their child is a bit better. We would like to know if children think it's ok to do research in emergencies without telling the parents or the child first.

Who can be involved?

We are interested to talking to children aged 7-<16 years old who have received emergency hospital treatment.

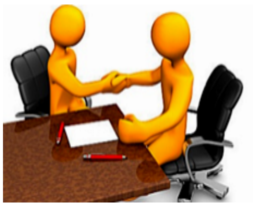

What is involved

We would like children to take part an interview with a researcher called Louise. Th can be at your home, on the phone, or at Alder Hey Children's Hospital; this is your choice. It will take about 45 minutes to an hour.

In the interview Louise will talk to your child about how research is done in emergencies. We would like to know what your child thinks about this type of research and find ways of involving children in decisions about emergency research in the future.

Following the study

We would like to give your child some vouchers (£10) as a thank you for participating in our research study and a certificate of participation.

Who is involved in the study?

The study is being organised by the Universit of Liverpool and has been approved by a research ethics committee (ref: 15/NW/0915).

If you would like to be involved please speak to your child and contact Louise sending her your contact details and age of the child/ young person so we can send out the correct participant information sheet (the information in these varies with the age of the child)
t: 0151 795 5326
E: consent@liv.ac.uk

And, check out our website:
http://www.liv.ac.uk/psychology-health-and-society/research/childrens-voices/about/

For peer review only

BMJ Open

CHILDREN’S VIEWS ON RESEARCH WITHOUT PRIOR CONSENT IN EMERGENCY SITUATIONS: A UK QUALITATIVE STUDY

Journal:	*BMJ Open*
Manuscript ID	bmjopen-2018-022894.R1
Article Type:	Research
Date Submitted by the Author:	27-Apr-2018

Complete List of Authors:	Roper, Louise; University of Liverpool, Institute of Psychology Health and Society<br>Sherratt, Frances C.; University of Liverpool, Department of Psychological Sciences<br>Young, Bridget; University of Liverpool, Department of Psychological Sciences<br>McNamara, Paul; University of Liverpool, Department of Child Health, Institute of Translational Medicine<br>Dawson, Angus; The University of Sydney Camperdown Campus<br>Appleton, Richard; Alder Hey Children's NHS Foundation Trust, Neurology<br>Crawley, Esther; University of Bristol, Bristol Medical School<br>Frith, Lucy; University of Liverpool, Department of Health Service Research<br>Gamble, Carrol; The University of Liverpool, Biostatistics<br>Woolfall, Kerry; University of Liverpool, Institute of Psychology Health and Society

**Primary Subject Heading**:	Emergency medicine
Secondary Subject Heading:	Intensive care, Ethics, Patient-centred medicine, Qualitative research
Keywords:	ACCIDENT & EMERGENCY MEDICINE, Paediatric intensive & critical care < INTENSIVE & CRITICAL CARE, MEDICAL ETHICS, QUALITATIVE RESEARCH, Consent

SCHOLARONE™
Manuscripts

TITLE: CHILDREN'S VIEWS ON RESEARCH WITHOUT PRIOR CONSENT IN EMERGENCY SITUATIONS: A UK QUALITATIVE STUDY

Louise Roper[1], Frances Sherratt[1], Bridget Young[1] , Paul McNamara[2], Angus Dawson[3], Richard Appleton[4], Esther Crawley[5], Lucy Frith[1], Carrol Gamble[6], Kerry Woolfall[1].

Corresponding author: Dr Kerry Woolfall, Institute of Psychology, Health & Society, University of Liverpool, Liverpool, UK, K.Woolfall@liverpool.ac.uk, Tel: 0151 794 4634

Affiliations:

1. Institute of Psychology, Health & Society, University of Liverpool, Liverpool, UK

2. Department of Child Health, Institute of Translational Medicine, University of Liverpool, Liverpool, UK

3. School of Public Health, University of Sydney, Sydney, Australia

4. Neurology Department, Alder Hey Children's Hospital, Liverpool, UK

5. Bristol Medical School, University of Bristol, Bristol, UK

6. Clinical Trials Research Centre (CTRC), University of Liverpool, Liverpool, UK

Key words: Accident & Emergency Medicine, intensive & critical care, medical ethics, qualitative research, consent

Word count: 3959

For peer review only

ABSTRACT

Objectives: We explored children's views on research without prior consent and sought to identify ways of involving children in research discussions.

Design: Qualitative interview study.

Setting: Participants were recruited through a UK children's hospital and online advertising.

Participants: 16 children aged 7-15 years with a diagnosis of asthma (n=14) or anaphylaxis (n=2) with recent (< 12 months) experience of emergency care.

Results: Children were keen to be included in medical research and viewed research without prior consent as acceptable in emergency situations if trial interventions were judged safe. Children trusted that doctors would know about their trial participation and act in their best interests. All **felt that children should be informed about the research following their recovery and involved in discussions with a clinician or their parent(s) about the use of data** already collected, as well as continued participation in the trial (if applicable)**.** Participants suggested methods to inform children about their trial participation including an animation.

Conclusions: Children supported, and were keen to be involved in, clinical trials in emergency situations. We present guidance and an animation that practitioners and parents might use to involve children in trial discussions following their recovery.

Strengths and limitations of this study

- This is the first study to explore the views and acceptability of research without prior consent (RWPC) among children with experience of receiving emergency treatment for life threatening conditions.
- We provide guidance and an animation to assist trial practitioners in involving children in RWPC discussions in collaboration with parents.
- Participants did not have personal experience of RWPC so findings were limited to children's views on hypothetical scenarios.
- Participants lived in areas with varied levels of social deprivation. Despite attempts to further maximize sample diversity by use social media, the majority of children were recruited from one UK children's hospital.

For peer review only

INTRODUCTION

Informed consent for participation in research is a key principle of good clinical practice and protects an individual's right to autonomy[1 2]. Children under the age of 16 are not legally permitted to give consent for their participation in clinical trials of drug treatments[3]. Nevertheless, the United Nations Convention on the Rights of the Child[4], international legislation[5] and research guidelines[6] recommend that children should be involved in decision making processes in a way that is age appropriate to the child and the context of their family. The term assent is widely used and refers to when a child's wishes are taken into consideration in research decision making. It is recommended that assent is sought from children before they take part in research, where informed consent is not possible, although the role of assent has been criticized for being unclear in research discussions with children.[7 8]

Neither informed consent nor assent are appropriate or feasible in acute paediatric emergency situations[9], as children are incapacitated and the time taken to discuss research with parents could delay lifesaving treatments[10 11]. To facilitate crucial clinical research and to advance evidence-based emergency medicine[12], changes to international legislation have allowed doctors to enter patients into clinical research without prior consent (RWPC)[3 13-16]. In the United Kingdom, RWPC, also known as deferred consent, involves approaching parents or legal representatives after the investigational treatment has been given and seeking permission for the use of data already collected, as well as any continued participation in the trial[17].

Guidance[18] and legislation[15] state that where possible, children should be involved in RWPC discussions once they have recovered. However, practitioners report that RWPC discussions with parents take place at the bedside, shortly after the emergency situation has passed and that children are rarely involved at this point because they too sick[19]. It is therefore likely that most children randomised into UK trials conducted in time critical situations have no knowledge or involvement in the decision about their participation in emergency research.

Despite growing literature on the views of parents and practitioners regarding paediatric trials conducted in life threatening situations[18 20 21], the opinions of children have not been investigated. This is an important omission as it is they who are most directly affected in this process. We aimed to explore children's perspectives on paediatric emergency research, including their views on the acceptability of RWPC and ways of involving them in RWPC discussions when they have recovered.

For peer review only

METHODS

Study design and setting

We chose a qualitative interview design to help us explore children's views in a flexible, child friendly way, and to provide insight into children's perspectives on RWPC[22 23]. We recruited children for interviews through a children's hospital in the North West of England, supplemented by online-advertising to promote sample diversity.

Patient and Public Involvement

Six members of the National Institute for Health Research (NIHR) Young Person's Advisory Group (YPAG) joined our 'Voices Advisory Group' to inform all aspects of the study. This included their collaboration in the development of recruitment materials, a topic guide, which included age-appropriate explanations of key topics (e.g. RWPC; see Supplementary file 1) and vignette (see Supplementary file 2). KW and LR discussed initial findings with the Voices Advisory Group to assist analysis and interpretation. Members of the Voices Advisory Group also presented the findings with KW to a wide audience (at conferences, and University events)

Selection of participants

Children were eligible if they: were aged 7-15 years; had written parental/legal representative consent to participate; had received emergency treatment in hospital in the previous 12 months; had capacity to assent and an acceptable standard of English.

Two clinical practitioners identified and approached parents and children who met the inclusion criteria, explained the aims of the study and provided age appropriate information sheets. Parents of the children who wished to participate completed a contact form, which was posted to LR. For online recruitment, FS contacted relevant support groups and asked them to place a study advertisement (Supplementary file 3) on their Facebook page, Twitter account, or website. The advert included details of how children or parents could contact the study team to register interest in participation.

Interview design and *conduct*

LR, a health psychologist arranged interviews either in the family's home, hospital or by telephone depending on participant preference. Before interviews LR explained the study, referring to the information sheets and consent/assent forms provided. Consent and assent forms were completed by parents and children before the interview began.

We began interviews by showing children the Nuffield Council's animation[6] on a laptop or iPad to help set the scene and explain clinical research participation in a child-friendly way. No child

For peer review only

had previous experience of RWPC, although they all had experienced at least one episode of receiving emergency care. We presented RWPC neutrally so that children could give their views freely. We also reassured children that there were no right or wrong answers.

LR conducted all interviews. Recruitment and interviews were discontinued when data saturation was reached, i.e. the point where no new themes were discovered in the analysis[24]. Interviews were audio-recorded, transcribed verbatim, and then anonymised. Participants were given a £10 shopping voucher and a certificate of participation as appreciation for their time.

Data analysis

Our approach to data analysis was interpretive and iterative, referring back and forth between developing analysis and gathering new data for evidence of children's views on research in emergency situations, the acceptability of RWPC and ways of involving children in discussions about research in this context[25 26]. A key consideration during analysis was that of catalytic validity, whereby findings should be relevant to future research and practice[27]. NVivo software[28] assisted data organisation. Several members of the research team (KW, LR, FS, BY) contributed to the analysis to ensure analytical rigor[26 29]We present selected interview quotations (with pseudonyms) that illustrate research themes across a range of participants. Where quotes have been shortened for brevity or to remove identifiable information, omitted text is marked with '…', and explanatory text is in brackets.

RESULTS

Participants

Sixteen children (aged 7-15 [mean 10.2] years) were interviewed. At the children's request, parents were present for most the interviews (n = 12/16, 75%). Most participants had chronic asthma (n = 14/16, 87%) and were recruited by a paediatric doctor (PM) in an outpatient clinic or hospital ward (n=2/16, 13%). Two participants suffered with anaphylaxis and were recruited via Facebook. Interviews averaged 34 minutes in length, ranging from 21-57 minutes (Table 1). Participants' postcodes indicated that 9/16 (56%) participants lived in areas of high deprivation (Indices of Multi Deprivation [IMD] decile 1-3), 5/16 (31%) lived in areas of moderate deprivation (IMD decile 4-6), whilst 2/16 (13%) children lived in the least deprived (IMD decile 7-10) areas of the UK[30].

For peer review only

Pseudonym	Gender	Age	Health condition	Parent present
Chloe	Female	12	Asthma	Yes
Charlie	Male	7	Asthma	Yes
James	Male	11	Asthma	Yes
Emily	Female	15	Asthma	No
Niamh	Female	15	Asthma	Yes
Kaitlin	Female	8	Asthma	Yes
Joseph	Male	7	Asthma	Yes
Josh	Male	11	Asthma	Yes
Mia	Female	9	Asthma	Yes
Daisy	Female	7	Asthma	Yes
Kevin	Male	8	Asthma	Yes
Patrick	Male	9	Asthma	No
Lola	Female	7	Asthma	Yes
Ryan	Male	8	Asthma	Yes
Tom	Male	15	Anaphylaxis	No
Tilly	Male	15	Anaphylaxis	No

Table 1: Participant and interview details

Support for RWPC, but only if a trial treatment was judged safe

All children voiced support for RWPC in emergency situations where a child's life was in danger, such as during an asthma attack or seizure. They explained that while parents and children should usually be asked for "permission" before the research takes place, RWPC was acceptable in emergency research as it is important that doctors were able to give trial medicines without any delay in this situation.

> **Chloe, aged 12:** *most of the time (in research) you should ask the permission from the parents and the children… I think they can do that (research) without asking for permission because if it's an emergency and you have to give the medicine.*

The researcher (and the vignette of Daniel, see S3), informed children that only medicines that were believed to be safe would be used in paediatric trials. Children viewed this information as important,

For peer review only

indeed, many stated that RWPC was only acceptable if doctors thought the treatment being tested was safe.

> **Josh, aged 11:** *If, say, like you're giving them a drug that hasn't been proven to have high probability of being safe, I don't think it would be good to give it them, but if it was probably going to be safe for them, I think they'd be fine.*

One child supported RWPC in the hope that the treatment being tested would help children to recover more quickly, and to inform future paediatric medicine. However, he warned that parents may have a different perspective and may be angry to find out that their child had been entered into a trial without informed consent if it turned out that the treatment was not effective.

> **Researcher:** *So what do you think about doing research without asking parents or children when they're very sick?*
>
> **James aged 11:** *I think the parents would be a bit ticked off like because they just want to get their child better, like I know my mum definitely would be…I would like it because if that drug did work on the off-chance then I would want to tell people* (other children with chronic health conditions).

Misconceptions and misunderstandings

Many children appeared to understand that trials *"try to see if"* the medicine being investigated makes children *"better or not"* (Lola aged 7). However, a few children appeared to hold a misconception that research participation would have similar benefits to clinical care. For some children this misconception seemed to be linked to the involvement of clinicians in the trial. Children stated they would not be upset about being entered into a RWPC trial as long as *"the doctors know"* (Emily aged 15) about their participation. As the quotes below suggest, children trusted clinicians to have their best interests at heart and-wanted assurance that doctors and nurses would know about, and have approved, their involvement in a trial.

> **Researcher:** *So what if they gave you a new type of nebuliser and not told you? Do you think if it's that type of thing you should have been asked* (about beforehand)*, or do you still think it's* (RWPC is) *okay?*
>
> **Emily aged 15:** *The doctors know* (about being given a trial intervention)*?*
>
> **Researcher:** *The doctors yes.*

Emily aged 15: *Oh yes, definitely.*

Researcher: *Would you be upset that they have tried something on you and not asked you first?*

Ryan aged 8 *No.*

Researcher: *Why is that?*

Ryan aged 8: *Because the doctors and the nurse, they know.*

Others believed that within a trial, children would receive the 'right' treatment, whist another implied that RWPC would only be acceptable if the intervention was effective:

Daisy aged 7: *"He'll (Daniel) get the right medicine to make him feel better.*

Tilly aged 14: *I don't see the problem if it's going to stop it* (anaphylaxis).

Why children want to be informed and involved in RWPC discussions

Children felt strongly they should be involved in RWPC discussions when they were feeling better. Several asked rhetorically, "Why shouldn't we be told?" when asked about their participation in research. Irrespective of their age, children reasoned they should be informed about the treatment received in a trial because *"it's going to impact on their body"* (Tilly, aged 14) not their parents' bodies. Indeed. Tilly added that not being informed about their participation in a trial would *"feel like I'd been sort of left out, like my opinions and views sort of, I don't get to express them"* suggesting that not being included in RWPC discussion would deny children their voice and the opportunity to have a say in their healthcare treatment.

A few children described wanting to be informed about their involvement in research and the trial results to *"know what the trial had proved"* (Chloe aged 12) for their own chronic health condition. They expressed a wish to help inform future knowledge about treatments for other children by finding "*out about new medicines on the computer that are better than other ones and then they could give us it and then it might make us better."* (Joseph aged 7).

Finally, two young boys (<12 years) wanted to know about their participation because they viewed medical research as exciting. For them children should be informed about their participation

For peer review only

in research because "*it could be fun to take part in stuff*" (Joseph, aged 7) and *"it's quite cool to be part of like a scientific medical thing"* (Josh, aged 11).

Who should explain RWPC to children?

Most children commented that the best person to explain that they had participated in a study while they were ill would be a doctor or nurse as they would be the most knowledgeable.

> **Tilly, aged 14:** *if it's a nurse who knows all about it and then also has studied kids, they can then help with that as well, help them do it, understand what's happened and go through it. But I don't think I'd listen if my mum told me. I would want it more, the medical advice really.*

> **Kaitlin, aged 8**: *By the doctor … because he can explain it more.*

Some envisaged they would have questions about the research which they would like a doctor or nurse to answer, such as: "*How could it affect me? What would be like the aftermath of it?"* (James aged 11), "*What could the risks be?"* (Joseph aged 7) as the doctor or nurse would be best placed to answer.

Although some children said they *"wouldn't mind who told"* (Chloe aged 12) them about their participation in a trial, a few younger children stated that they would prefer to be informed by their mothers because it may *"feel a bit awkward"* talking to the doctors and its *"easier with my mum"* (Mia aged 9).

Children's role in decisions about the use of their information

Children varied in their views about the role of children in RWPC decisions when they were feeling better. Younger children were typically happy for their parents (mainly mothers) to make the final decision about the use of their information in a trial.

> **Kevin aged 8**: *Up to my mum.*

> **Joseph, aged 7**: *I would listen to my mum and the doctors because older people can sometimes be cleverer than younger people than them, because they've had more years to learn about stuff.*

For peer review only - http://bmjopen.bmj.com/site/about/guidelines.xhtml

Participants of all ages echoed this by commenting that children younger than themselves, *"don't really understand […] what the research is about"* (Chloe aged 12) and *"might be a bit too young"* (Joseph aged 7) to make such decisions, and that for such children it should be a parent's role to make a decision about the use of their information for research purposes.

Similarly, some children stated that they would accept their parents' decision, even if it was at odds with their own views, whilst others wanted to share in decision-making with their mothers.

> **Emily aged 15:** *My little brother, he's 11 and I think he's sensible so he'd probably choose the right thing. But if you're a bit younger I think it should be up to your mum.*
>
> **James, aged 11:** *If mum said no and I said yes, I would just listen to mum.*
>
> **Researcher:** *What about if you wanted to be in it but mum said no, then who should (the doctor) listen to?*
> **Chloe, aged 8:** *Both of us.*

In contrast, a few teenage participants felt that *"they should be able to actually make a medical choice for themselves because it's impacting them in the end"* (Tilly aged 14). Therefore, their decision about the use of their data and involvement in any follow up, should override, or at least have greater value, than their parents' decisions.

> **Researcher:** *What if your mum says yes, that's fine and you say no? Then who should be in charge?*
> **Emily aged 15:** *Me*

Child friendly resources to help communicate RWPC: introducing the 'You took part in Research animation'

Finally, we sought participants' suggestions on the most appropriate resources to help to engage with children in RWPC discussions when they were feeling better. Participants emphasized the need for a face to face discussion so doctors or nurses could *"explain it to you, or give you a leaflet, then…you can ask questions in person"* (Chloe aged 12). While a few suggested that leaflets, websites and picture books would be useful in explaining RWPC, most favoured an online animation that could be used either in hospital as part of a face to face discussion, or *"at some point when I*

For peer review only

was at home" (Tom aged 13) to *"make sure they understand everything properly"* (Josh aged 11) to explain RWPC.

> **Patrick aged 9:** "*because some doctors don't know how far to explain stuff when they are particularly young kids. So even the doctors to have on their phone or their iPad and go this is what we've done, and then they watch it and then they explain a bit more then*".

The findings of this study encouraged the study team to develop an animation with the help of the Voices Advisory Group and three of interviewees in the current study who wished to help with this. The 'You took part in research' animation available to view on YouTube (https://www.youtube.com/watch?v=_Fs1yUxeBFQ), focuses on explaining why consent could not be sought before a child took part in research.

DISCUSSION

We believe this is the first study to explore the views and acceptability of RWPC among children with experience of receiving emergency treatment in hospital. The children we interviewed supported RWPC in the hope that such research would contribute to developments in children's emergency medicine for the benefit of themselves and other children. However, RWPC was only acceptable to children where trial interventions were believed by the clinicians caring for them to be safe. This finding also echoes previous literature on therapeutic misconceptions about trial participation amongst adults[31], as some children held the misconception that research participation would have similar benefits to clinical care. Others appeared to trust that doctors would know about their trial participation and act in their best interests. Others believed that within a trial, children would receive the 'right', treatment. Such findings suggest that trial information materials and practitioner RWPC discussions should clearly explain that doctors don't know which intervention is the best, which is why a trial is needed. This information should be supplemented with details of other treatments given (if applicable) and any additional monitoring that occurred.

This study has strengths and limitations. This is the first study to explore the views and acceptability of RWPC among children with experience of receiving emergency treatment in hospital. We strengthened our qualitative sampling by conducting interviews until no new relevant knowledge was obtained from new participants (data saturation). All participants had a chronic health condition and experienced multiple hospital admissions for emergency lifesaving treatments. However, our findings do not reflect the views of acutely ill children admitted to hospital for the first time. Participants did not have personal experience of emergency medicine research so findings

For peer review only - http://bmjopen.bmj.com/site/about/guidelines.xhtml

were limited to children's views on hypothetical scenarios about RWPC. Finally, the study findings are mainly limited to the views of children recruited from an asthma clinic in one UK children's hospital. Nevertheless, there was variation in the sample, as children lived in areas with differing levels of social deprivation. Despite the successful use of websites and social media advertising to recruit parents to similar qualitative studies[21 32], only two children responded to social media advertisement. The low response to this indicates social media such as Facebook and Twitter may not be an effective route for recruiting children to research. Placing adverts on social media more commonly used by children (e.g. Snapchat)[33] may have been more successful.

Children's support of research without prior consent in emergency situation reflects the findings of previous studies on this topic involving trial practitioners and parents[18 19 21 32 34]. Where studies[18 32 35] have shown that parents can have initial negative responses to RWPC, children did not appear to be shocked or surprised at this alternative to giving informed consent before the research intervention. This may reflect their lack of familiarity with standard research consent processes, or indeed, their lack of previous involvement in decisions about their healthcare. Our findings suggest that children trust doctors and parents to make appropriate decisions about their participation in research when they are unable to do so themselves. However, children were clear that when they were feeling better, adults (particularly mothers and doctors) should inform them of their trial participation and discuss the use of their data.

Children in our study acknowledged the important role that children, parents and doctors played in research decision making. This reflects guidelines recommending that decisions about research should involve discussion and information exchange between children, their parents and doctors[6 12 36-39]. Some described the prospect of research participation as exciting and fun, whilst others, seemed to want the satisfaction of knowing if they had contributed to research that might improve future treatments for both themselves and others. Children wanted to be involved in decisions regarding their own healthcare [40 41]. Younger children were happy for their parents to take the lead or a shared role in research decision-making. A few teenagers felt their views should carry greater weight than their parents suggesting that practitioners should be aware of the possibility that teenagers may wish to take a lead in such decisions. As previous literature [19 21 32] and Voices study findings all show support for RWPC, we do not anticipate divergence in child and parent views on consent in this situation; mainly because the intervention has already been given and children have commonly recovered when research discussions take place.. The challenge is ensuring children are given the opportunity to be (retrospectively) informed about their research participation and actively involved in research discussions and decision making with their parents.

For peer review only - http://bmjopen.bmj.com/site/about/guidelines.xhtml

We acknowledge there may be practical barriers to involving children in RWPC discussions, as they may not be well enough, or have the capacity to be involved before discharge from hospital. Nevertheless, our findings[19] indicate that when they have recovered children are not given the opportunity to know they have participated in research or a say in whether their data continue to be used in research. Based on the accounts of the children we interviewed, this is unlikely to be acceptable to children who participate in trials to evaluate interventions used in emergency care. As one child we interviewed eloquently stated, *"it's not impacting their life, it's impacting your own"* (Tilly, aged 15).

Box 1 (guidance to inform and involve children in RWPC discussions) provides an update to CONNECT study RWPC guidance[42], which aims to assist trial practitioners in involving children in RWPC discussions in collaboration with parents. *To respect the wishes of children we encourage trial staff to involve them in trial discussions in hospital if they are well enough. The 'You took part in research' animation can be used to support these discussions. If this is not possible before children are discharged from hospital. Age appropriate information can be provided to facilitate family discussions about trial participation at home, including contact details if children wish to discuss their participation with the trial team Whilst our focus in this paper has been children (under 16 years) we believe our findings also relate to trial discussions with young people (16-18 years).*

Children should be given an opportunity to be involved in making decisions about the use of their data in the trial and their continued enrolment if they have capacity.

• When children cannot be involved in trial consent discussions due to a lack of capacity, provide age appropriate trial information (such as a leaflet or website) to parents for children to read when they have recovered.

• Include a link to the 'You took part in research animation' https://www.youtube.com/watch?v=_Fs1yUxeBFQ) to help explain why consent could not be sought before a child took part in research.

• Provide research team contact details so that parents or children can discuss any aspect of the trial at a later date.

Box 1: Guidance to inform and involve children in RWPC discussions

Further research is needed to explore the experiences of children with first-hand experience

For peer review only

of RWPC and associated discussions.

Transparency statement

The lead author affirms that the manuscript is an honest, accurate, and transparent account of the study being reported; that no important aspects of the study have been omitted; and that any discrepancy from the study as originally planned has been explained. The report is independent research supported by the National Institute for Health research (Senior Research Fellowship, Prof Esther Crawley, SRF-2013-06-013). The views expressed in this publication are those of the authors and not necessarily those of the NHS, the National institute for Health Research or the Department of Health.

Author contributions:
KW conceived the study. KW, BY, LR, and FS designed the study. PM, LR and FS recruited children to the study. LR conducted the interviews. LR, FS and KW analysed the data. KW and LR drafted the paper. KW, LR, FS, BY, PM, AD, RA, EC, LF and CG contributed to revisions and approved the final draft. KW takes responsibility for the paper as a whole. We would like to thank our Young Person's Advisory Group (YPAG) for their valuable insights into the study design, conduct and interpretations of results; as well as the children who took part in the study and helped develop the animation. We would also like to thank Beth Morris and Paige Karadag for their comments on the final draft.

Funding statement
The study was funded by the MRC Hubs for Trials Methodology Research (project R42) and supported by Wellcome Trust ISSF award.

Ethics statement
A National Health Service Research Ethics Committee (15/NW/0915) provided ethical approval.

Data sharing statement No additional data are available

References

1. Nelson RM, Beauchamp T, Miller VA, et al. The Concept of Voluntary Consent. *The American Journal of Bioethics* 2011;11(8):6-16. doi: 10.1080/15265161.2011.583318
2. Beauchamp T, Childress J. Principles of Biomedical Ethics. Oxford: Oxford University Press 2001.
3. Legislation.gov.uk. Medicines for Human Use (Clinical Trials) Regulations 10: SI 2004/1031 2004.
4. United Nations. Convention on the Rights of the Child. 1989. http://www.ohchr.org/Documents/ProfessionalInterest/crc.pdf (accessed 3rd June 2016).
5. legislation.gov.uk. Children Act 1989, 1989.
6. Nuffield Council on Bioethics. Children and clinical research: ethical issues. London: Nuffield Council on Bioethics, 2015.
7. Baines P. Assent for children's participation in research is incoherent and wrong. *Archives of Disease in Childhood* 2011;96(10):960-62. doi: 10.1136/adc.2011.211342

For peer review only

8. Madden L, Shilling V, Woolfall K, et al. Questioning assent: how are children's views included as families make decisions about clinical trials? *Child: Care, Health and Development* 2016;42(6):900-08. doi: 10.1111/cch.12347
9. Eltorki M, Uleryk E, Freedman SB. Waiver of informed consent in pediatric resuscitation research: a systematic review. *Acad Emerg Med* 2013;20(8):822-34. doi: 10.1111/acem.12180 [published Online First: 2013/09/17]
10. DIRECTIVE 2001/20/EC OF THE EUROPEAN PARLIAMENT AND OF THE COUNCIL of 4 April 2001on the approximation of the laws, regulations and administrative provisions of the Member States relating to the implementation of good clinical practice in the conduct of clinical trials on medicinal products for human use. 2001; L 121/34. http://www.eortc.be/services/doc/clinical-eu-directive-04-april-01.pdf.
11. Roberts I, Prieto-Merino D, Shakur H, et al. Effect of consent rituals on mortality in emergency care research. *The Lancet* 2011;377(9771):1071-72. doi: http://dx.doi.org/10.1016/S0140-6736(11)60317-6
12. Brierley J, Larcher V. Emergency research in children: Options for ethical recruitment. *Journal of Medical Ethics* 2011;37(7):429-32.
13. Kompanje EO, Maas AR, Menon D, et al. Medical research in emergency research in the European Union member states: tensions between theory and practice. *Intensive Care Medicine* 2014;40(4):496-503. doi: 10.1007/s00134-014-3243-6
14. Iwanowski P, Budaj A, Czlonkowska A, et al. Informed consent for clinical trials in acute cirinary syndromes and stroke following the European clinical trials directive: investigators' experiences and attitudes. *Trials* 2008;9(45):1-6.
15. Legislation.gov.uk. The Medicines for Human Use (Clinical Trials) and Blood Safety and Quality (Amendment) Regulations 2008 941. 10. 2008
16. Council of the European Union. Proposal for a Regulation of the European Parliament and of the Council on Clinical trials on medicinal products for human use, and repealing Directive 2001/20/EC. 2013. (accessed 29/01/14).
17. Fost N, Robertson J. Deferring Consent with Incompetent Patients in an Intensive Care Unit. *IRB: Ethics and Human Research* 1980;2(7):5-6. doi: 10.2307/3564363
18. Woolfall K, Frith L, Dawson A, et al. 15 minute consultation: an evidence-based approach to research without prior consent (deferred consent) in neonatal and paediatric critical care trials. *Archives of disease in childhood - Education & practice edition* 2015;101(1):49-53. doi: 10.1136/archdischild-2015-309245
19. Woolfall K, Frith L, Gamble C, et al. How parents and practitioners experience research without prior consent (deferred consent) for emergency research involving children with life threatening conditions: a mixed method study. *BMJ Open 2015*;5(9):e008522 doi: 10.1136/bmjopen-2015-008522
20. Lyttle MD, Gamble C, Messahel S, et al. Emergency treatment with levetiracetam or phenytoin in status epilepticus in children—the EcLiPSE study: study protocol for a randomised controlled trial. *Trials* 2017;18(1):283. doi: 10.1186/s13063-017-2010-8
21. Woolfall K, Young B, Frith L, et al. Doing challenging research studies in a patient-centred way: a qualitative study to inform a randomised controlled trial in the paediatric emergency care setting. *BMJ Open* 2014;4(5) doi: 10.1136/bmjopen-2014-005045
22. Murphy E, Dingwall R. Qualitative methods in health services research. *Health services research methods* 1998:129 - 38.
23. Morrow V. Using qualitative methods to elicit young people's perspectives on their environments: some ideas for community health initiatives. *Health Education Research* 2001;16(3):255-68. doi: 10.1093/her/16.3.255
24. Guest G, Bunce A, Johnson L. How Many Interviews Are Enough?: An Experiment with Data Saturation and Variability. *Field Methods* 2006;18(1):59-82. doi: 10.1177/1525822x05279903

25. Strauss A, Corbin J. Basics of qualitative research: Techniques and procedures for developing grounded thoery 2nd edition. Thousand Oaks, CA: Sage 1998.
26. Mays N, Pope C. Assessing quality in qualitative research. *BMJ* 2000;320(7226):50-52. doi: 10.1136/bmj.320.7226.50
27. Stiles WB. Evaluating qualitative research. *Evidence Based Mental Health* 1999;2(4):99-101. doi: 10.1136/ebmh.2.4.99
28. NVivo qualitative data analysis Software [program], 2012.
29. Tong A, Sainsbury P, Craig J. Consolidated criteria for reporting qualitative research (COREQ): a 32-item checklist for interviews and focus groups. *International Journal for Quality in Health Care* 2007;19(6):349-57. doi: 10.1093/intqhc/mzm042
30. Department for Communities and Local Government. File 7: all ranks, decile and scores for the indices of deprivation, and population denominators. In: Official Statistics, ed. London, 2015.
31. Lidz CW, Appelbaum PS, Grisso T, et al. Therapeutic misconception and the appreciation of risks in clinical trials. *Social Science & Medicine* 2004;58(9):1689-97. doi: https://doi.org/10.1016/S0277-9536(03)00338-1
32. O'Hara CB, Canter RR, Mouncey PR, et al. A qualitative feasibility study to inform a randomised controlled trial of fluid bolus therapy in septic shock. *Archives of Disease in Childhood* 2017 doi: 10.1136/archdischild-2016-312515
33. Ofcom. Children and Parents: Media Use and Attitudes Report. London, 2017.
34. Gamble C, Hickey H, Snape D, et al. Parental attitudes to deferred consent in randomised trials in emergency resuscitation: a postal survey of Meningitis Research Foundation members. *Submission pending* 2010
35. Woolfall K, Frith L, Gamble C, et al. How parents and practitioners experience research without prior consent (deferred consent) for emergency research involving children with life threatening conditions: a mixed method study. *BMJ Open* 2015;5(9) doi: 10.1136/bmjopen-2015-008522
36. Modi N, Vohra J, Preston J, et al. Guidance on clinical research involving infants, children and young people: an update for researchers and research ethics committees. *Archives of Disease in Childhood* 2014 doi: 10.1136/archdischild-2014-306444
37. Nuffield Council on Bioethics. Children and clinical research: ethical issues. London, 2015.
38. Paediatrics RCo, Committee CHEA. Guidelines for the ethical conduct of medical research involving children. *Archives of Disease in Childhood* 2000;82(2):177-82. doi: 10.1136/adc.82.2.177
39. Helsinki WEU-Do. World Medical Association Declaration of Helsinki. In: Assembly WG, ed. Fortaleza, Brazil, 2013.
40. Moore L, Kirk S. A literature review of children's and young people's participation in decisions relating to health care. *J Clin Nurs* 2010;19(15-16):2215-25. doi: 10.1111/j.1365-2702.2009.03161.x [published Online First: 2010/07/28]
41. Shilling V, Williamson PR, Hickey H, et al. Processes in recruitment to randomised controlled trials of medicines for children (RECRUIT): a qualitative study. *Health Technol Assess* 2011;15(15):1-116. doi: 10.3310/hta15150 [published Online First: 2011/03/30]
42. Woolfall K, Frith L, Dawson A, et al. Research without prior consent (deferred consent) in trials investigating the emergency treatmemt of critically ill children: CONNECT study guidance. Liverpool: University of Liverpol, 2015.

For peer review only

For peer review only

Supplementary file 1: Description of RWPC in interviews

Normally, when doctors want to do research to find out the best medicine to give children to make them better they usually speak to mums, dads and children themselves to see if they would like to take part in the research. This is just to check that they are happy with the research before a medicine is given. But when children are very poorly and need emergency medicine- like in the back of an ambulance- there is no time to speak to anyone about the research. Doctors have special permission to give the medicine without asking parents and children first.

(Check child understands at this point and use drawing if necessary to assist understanding)

The reason this is allowed to give medicine as part of research without asking first is so that doctors can find out which are the best medicines to use to treat children when they are very sick. If they tried to speak to parents first it would mean that children wouldn't be given the medicine they need at the right time.

For peer review only

Supplementary file 2: vignette used in interviews

A little boy called Daniel had an illness which meant he sometimes had to go to hospital to be helped by the doctors and nurses to get better. The doctors asked Daniels mum and dad whether they could give him some different medicine to see if it was better than the medicine he usually got when he was unwell. They wanted to see what happened to Daniel when he got the new medicine and wanted to write extra information down about the new medicine. The new medicine was safe, but the doctors didn't know whether it was better or the same as the medicine Daniel usually got. The doctors decided that because Daniel was feeling well again and had had some rest, they wanted to explain to Daniel about the different medicine and the reason why they wanted him to take part. Daniel is the same age as you.

For peer review only

Voices Project– Children’s Views

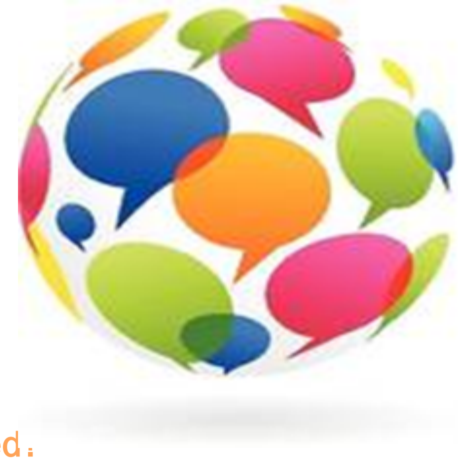

WOULD YOUR CHILD LIKE TO TAKE PART ?

What is the study about?

We are doing this study to find out what children think about research in emergency situations (such as in A&E).

When children are very sick it is important to give them medicine quickly. This means that there is no time to speak to parents or children about taking part in research before the emergency medicine is given. The doctors give the treatment first, and then speak to the parents about the study when their child is a bit better. We would like to know if children think it’s ok to do research in emergencies without telling the parents or the child first.

Who can be involved?

We are interested to talking to children aged 7-<16 years old who have received emergency hospital treatment.

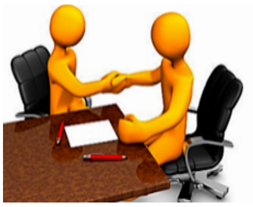

What is involved?

We would like children to take part an interview with a researcher called Louise. Th can be at your home, on the phone, or at Alder Hey Children’s Hospital; this is your choice. It will take about 45 minutes to an hour.

In the interview Louise will talk to your child about how research is done in emergencies. We would like to know what your child thinks about this type of research and find ways of involving children in decisions about emergency research in the future.

Following the study

We would like to give your child some vouchers (£10) as a thank you for participating in our research study and a certificate of participation.

Who is involved in the study?

The study is being organised by the Universit of Liverpool and has been approved by a research ethics committee (ref: 15/NW/0915).

If you would like to be involved please speak to your child and contact Louise sending her your contact details and age of the child/ young person so we can send out the correct participant information sheet (the information in these varies with the age of the child)
t: 0151 795 5326
E: consent@liv.ac.uk

And, check out our website:
http://www.liv.ac.uk/psychology-health-and-society/research/childrens-voices/about/

For peer review only

Standards for reporting qualitative research checklist		
**No.**	**Topic**	**Page number in manuscript (section)**
S1	Title	1
S2	Abstract	2
S3	Problem formulation	3
S4	Purpose or research question	3 (introduction)
S5	Qualitative approach and research paradigm	4 (study design and setting)
S6	Researcher characteristics and reflexivity	4 (interview design and conduct)
S7	Context	4 (study design and setting)
S8	Sampling strategy	4 (selection of participants)
S9	Ethical issues pertaining to human subjects	14 (ethics statement)
S10	Data collection methods	4 (study design and setting) and 5 (interview design and conduct)
S11	Data collection instruments and technologies	4 (patient and public involvement) and 5 (interview design and conduct)
S12	Units of study	5 (participants)
S13	Data processing	5 (interview design and conduct)
S14	Data analysis	5 (data analysis)
S15	Techniques to enhance trustworthiness	5 (data analysis)
S16	Synthesis and interpretation	6-11
S17	Links to empirical data	6-11
S18	Integration with prior work, implications, transferability, and contribution(s) to the field	11 (discussion)
S19	Limitations	2 (strength and limitations)
S20	Conflicts of interest	14 (transparency statement)
S21	Funding	14 (funding statement)

For peer review only
